# Modern Sociology

**Published:** 1900-12-01

**Authors:** 


### Modern Sociology.

### SOME DANGEROUS TRADES.

### Gannister Disease.

We have received from Dr. C. L. Birmingham, M.D., a copy of an address which he delivered before the Sanitary Institute at its Congress at Southampton, in 1899, on "Gannister Disease."

The first question put by most persons will be, "What is gannister disease?" But that should be preceded by the question "What is gannister?" Gannister is a very hard flinty rock, which is found in the under clays of some coal-measures. It is found in the coalfields of Northumberland; near Wrexham, in Denbighshire, and between Penistone and Sheffield, about the Valley of the Don, in Yorkshire. Gannister is found in other places, but Dr. Birmingham says that the places named are the only ones where it has a high commercial value. Hard as adamant, gannister cannot be quarried by the ordinary processes, and blasting has to be resorted to. The blocks of stone, when taken from the quarry, are ground into a fine powder, which is then made into bricks and tiles. These bricks possess a power of withstanding a temperature so high that other bricks would give way under it. For this reason, gannister is used "almost universally," Dr. Birmingham says, for lining the bottoms of the Bessemer converters. Dr. Birmingham studied the conditions of the gannister industry in the Don Valley, and there became convinced that it had special dangers which justified its being regarded as a dangerous trade.

The danger is the old one—dust. Dust arises in the blasting; not only the dust caused by the blasting that is taking place at any given moment, but the dust of former blastings which is lying about, swirled about again by the shock of the new blast. This has never thoroughly settled before the miner goes in again to the mine to see to the removal of the stone. Insensibly he breathes some of this dust, and though it may be arrested in the upper air passages, an extra long breath or an extra amount of dust in the atmosphere may cause him to inhale enough to affect the bronchial membranes. The gannister rock being so hard and flinty, the dust of it is sharp as needles, and soon finds out and irritates any weak spot in the organism. The mechanical irritation caused by the dust leads to inflammation and ultimately to suppuration. The miner first suffers from bronchitis, but soon the disease spreads to the lungs, and the typical symptoms resemble phthisis. As Dr. Birmingham says, "The miner is an anæmic, enfeebled individual, breathing rapidly and with great difficulty, incapable of making any physical effort, and troubled with a perpetually hacking cough. He walks about his village an object of pity, 'waiting for his turn.' Perhaps he will have four years to wait, commonly he will have only four, more commonly he will have only two or three years to wait."

The dangers to which gannister grinders and brickmakers are subject are identical with those of the miners, but the mortality among the grinders shows that their part of the work is the most fatal, while among the brickmakers, probably because the powdered gannister is wet when they manipulate it, the health conditions do not seem to be so bad.

The industry is a small one, or perhaps special cognisance would have been taken of its dangers before now. In the district from which the author of the paper from which we quote obtained his data, there were 420 men and boys employed at it between the years 1894 and 1898. Rather more than half of these were miners. Of the remainder, 16 or 17 per cent. were labourers connected with the works who did not seem to suffer from any disease that could be traced to the handling of gannister. Dr. Birmingham's statistics are, therefore, drawn from the remaining 350 workers. He does not altogether rely on the registrar's death certificates for his inferences, although gannister disease is so far recognised that five deaths are stated to be due to it in the years 1891, 1892, and 1893. He thinks that the thirty deaths which are set down as being due to pulmonary phthisis might also be attributed to the dangers of working with gannister. He reckons that the annual death-rate among gannister workers is 22·29 per 1,000. He analyses the deaths in the different branches of the work thus:—

| | | |
|---|---|---|
| Gannister miners | ... 42·3 | per 1,000 per annum |
| " grinders | ... 179·8 | " " |
| " brickmakers | . 22·2 | " " |

"The wonder is," he says, "that men can be induced to work at a trade like gannister grinding, where there is the appalling high mortality rate of 179·8 per 1,000."

In 1891 an oro-nasal respirator was introduced among the gannister workers, and, says the gentleman from whom we quote, was "worn by the men when their masters were watching them." He describes it as "consisting of an ill-fitting, crudely-made mask of gauze, or perforated metal, to contain a moistened sponge and cover the nose and mouth." This respirator was clumsy and ill-fitting. It did not prevent dust from entering the nose and mouth, and it was a great inconvenience to the men when they were doing heavy work, such as using the pick or sledge-hammer. "As a matter of fact, few of the men wear their respirators except when doing quiet work, and many of them have holes made through the gauze to admit their tobacco-pipes." No severe penalty seems to be enforced against those who do not wear the respirator. The use of steam-jets over the grinders has been tried, but does not seem to have done much good. The only other preventive used has been the sprinkling of water carried into the pits in ordinary drinking bottles, where punching for blasting is going on.

Dr. Birmingham makes the following suggestions:— Only adult men between the ages of eighteen and sixty to be allowed to work at the gannister trade. Before commencing work the men should submit to a rigid medical examination, and be found to be absolutely free from all disease of the respiratory, cardiac, and renal systems. While engaged at the work, the men should be examined at least every three months, and not allowed to continue work if any evidence of bronchitis or other affection of the respiratory system be discovered. On the disappearance of such evidences they may be permitted to resume work, but should for six months subsequently be medically examined once a month. He recommends the provision of efficient protective respirators, "if such can be found," and the infliction of severe penalties for omitting to use them. Automatically acting machinery should be introduced wherever practicable, and a plentiful supply of water should be produced from a hose, both before and after all processes calculated to agitate dust. Ventilation should at all times be as efficient as possible. Finally, "frequent inspections should be made of both the surface and underground workings by a capable Government official." But is there no Government inspection of this industry? If not, why not? It is small, but should not on that account be omitted from supervision.

